# Tuning the optical properties of poly(o-phenylenediamine-co-pyrrole) via template mediated copolymerization

**DOI:** 10.1080/15685551.2018.1459078

**Published:** 2018-05-09

**Authors:** Sapana Jadoun, Liza Biswal, Ufana Riaz

**Affiliations:** a Materials Research Laboratory, Department of Chemistry, Jamia Millia Islamia, New Delhi, India

**Keywords:** Copolymers, polypyrrole, poly(o-phenylenediamine), chemical polymerization, fluorescence

## Abstract

Tailoring of conjugated monomers via copolymerization is a facile method to obtain tunable spectral, morphological and optical properties. To investigate the effect of copolymerization of pyrrole with o-phenylenediamine on the optoelectronic properties of the synthesized copolymers, the present work reports the synthesis of copolymers of o-phenylenediamine with pyrrole with varying mol ratios via chemical polymerization in methylene blue (MB) medium. Copolymerization was confirmed by Fourier transform infrared spectroscopy and ultraviolet-visible studies. Ultraviolet-visible spectroscopy revealed variation in the optical properties with the change in the monomer ratio. Fluorescence studies showed that the copolymer containing 80% poly(o-phenylenediamine) revealed highest quantum yield among all the copolymers. The emission color could therefore be tuned by careful selection of narrow band co-monomers, which could help in designing tunable fluorescence emitting materials for potential application in OLED devices.

## 1. Introduction

Conjugated polymer nanoparticles have attracted significant attention due to their wide applications in corrosion protection, LEDs, rechargeable batteries, sensors, optoelectronic devices etc. [1–4]. Synthesis of conjugated polymers like polyaniline (PANI) [3], polypyrrole (Ppy) [1], Poly(phenylenediamine) (POPD) [5,6] polythiophenes (PTHs) [7], poly(1-naphthylamine) (PNA) [8,9] have been extensively reported in literature. Copolymerization of conjugated polymers is one of the most assorted routes adopted to prepare custom-made low band gap polymers [10,11]. It also allows designing of functionalized moieties having synergistic properties of both monomers [12,13]. Copolymers of cyclodiborazane dithiafulvene [14], p-phenylene vinylenes [2], carbazole-quinoline, phenothiazine-quinoline [15,16], o-aminophenol [17], o-methoxyanilene [18,19], o-toluidine, 1-naphthylamine [20], ethyl aniline [21,22], carbazole [23–25] have been widely reported in literature.

Although several copolymers have been synthesized, till date no literature has been reported on the copolymerization of o-phenylenediamine and pyrrole using dye as a template medium. Soft-template method has been extensively investigated for the synthesis of conducting polymers which involves the creation of support structures for the growth of 1-D structures [26]. This technique is highly cost-effective for obtaining polymers with controlled architectures. Although surfactants have been widely reported to be used as soft templates, the use of azo-dye dyes to obtain self-assembled morphology has also been explored via the creation of azo-dye-oxidant complexes [27,28]. The 1-D polymer structure is formed on the surface of the fibers, which gradually degrades during reaction.

With the aim to explore the role of methylene blue dye as a soft template as well as a dopant for conducting polymers, the present work reports the copolymerization of pyrrole with o-phenylenediamine via chemical oxidative polymerization. The influence of this soft template on spectral, morphological and fluorescent properties was investigated using FT-IR UV-Visible and fluorescence studies. Results revealed that the copolymers exhibited the tendency to undergo random copolymerization which could be tuned to obtain the desired optoelectronic properties.

## 2. Experimental

### 2.1. Materials

o-phenylenediamine (C_6_H_8_N_2,_ molar mass: 108.14 g/mol, melting point: 98–102 °C, boiling point: 256–258 °C) (Sigma Aldrich, U.S.A.), pyrrole (C_4_H_5_ N, molar mass: 67.09 g/mol, melting point: −23 °C, boiling point: 129–131 °C) (Sigma Aldrich, U.S.A.), potassium dichromate (K_2_Cr_2_O_7,_ molar mass: 294.18 g/mol, melting point: 398 °C), methylene blue (C_16_H_18_ClN_3_S, molar mass: 319.85 g/mol) 1-methyl-2-pyrillidone (C_5_H_9_NO, molar mass: 99.13 g/mol, melting point: −24 °C, boiling point: 202 °C, density: 1.028 g/mL at 25 °C) (Sigma Aldrich, U.S.A.), Acetonitrile (CH_3_CN, molar mass: 41.05 g/mol, melting point: −48 °C, boiling point: 81–82 °C) (Sigma Aldrich, U.S.A.), Dimethyl sulfoxide (C_2_H_6_OS, molar mass: 78.13 g/mol, melting point: 19 °C, boiling point: 189 °C) (Sigma Aldrich, U.S.A.), Sulfuric acid (H_2_SO_4_, molar mass: 98.079 g/mol, melting point: 10 °C, boiling point: 337 °C)(Sigma Aldrich, U.S.A.), PVA((C_2_H_4_O)_x_, density: 1.19–1.31 g/cm^3^, melting point: 200 °C (392 °F; 473 K), boiling point: 228 °C (442 °F; 501 K))(Sigma Aldrich, U.S.A.), Lead acetate (Pb(CH_3_CO_2_)_4_, molar mass 443.38 g/mol)(Sigma Aldrich, U.S.A.) were used without further purification.

### 2.2. Synthesis of homopolymers of o-phenylenediamine and pyrrole in methylene blue(MB) medium

Methylene blue dye solution (10^−3^ M) was prepared by dissolving 0.015 g MB in 50 ml distilled water. Potassium dichromate (2.7203 g, 0.3698 mol) and (2.1924 g, 0.2981 mol) were dissolved in deionized water (25 ml) in a 250 ml conical flask. O-phenylenediamine (1 g, 0.1849 mol) was added to MB solution (50 ml) followed by the addition of K_2_Cr_2_O_7._ The colour of the solution changed from transparent dark yellow to intense greenish brown indicating rapid polymerization of the monomer [29]. The reaction mixture was then stirred on a magnetic stirrer for 3 h at 25 °C. The obtained poly(o-phenylenediamine) was kept in a deep freezer for 24 h at −5 °C then washed with distilled water and ethanol several times. The removal of chromate ion was ensured by testing the filtrate with lead acetate and acetic acid. The polymer was dried in a vacuum oven for 72 h at 100 °C to ensure removal of water and impurities. The polymer was designated as POPD. For the synthesis of polypyrrole, pyrrole monomer (0.5170 ml, 0.1490 mol) was added to MB solution (50 ml) followed by the addition of K_2_Cr_2_O_7_ solution drop by drop with the help of burette keeping the monomer: initiator ratio 1:1. The flask was then stirred on a magnetic stirrer for 3 h at 25 °C. The obtained polypyrrole was then kept in a deep freezer for 24 h at −5 °C. The precipitated polypyrrole was washed several times with ethanol and distilled water to remove chromate ion. The greyish polypyrrole powder was dried in a vacuum oven for 72 h at 100 °C to ensure complete removal of impurities, water and unreacted monomer. This homopolymer was designated as Ppy. The percent yield for POPD and Ppy was calculated 92.77 and 83.06 respectively.

### 2.3. Copolymerization of o-phenylenediamine and pyrrole

Synthesis of copolymers of o-phenylenediamine and pyrrole was carried out using 3 M ratios of OPD and Py, that is, 80/20, 50/50, 20/80 respectively. The reaction mixture was added to 250 ml Erlenmeyer flask containing 10^−3^ M MB medium. Potassium dichromate was added to reaction mixture drop by drop with the help of burette keeping monomer: oxidant as 1:1. The reaction mixture was then subjected polymerization for 3 h at 25⁰C with continuous stirring and was kept in deep freezer for 24 h at −5 °C. Afterwards the reaction mixture was washed several times with distilled water and ethanol and dried in a vacuum oven in 100⁰C for 72 h to ensure complete removal of water and impurities. These copolymers were designated as POPD/Ppy-80/20, POPD/Ppy-50/50, POPD/Ppy-20/80. The percent yield obtained was 90.01, 87.3 and 84 respectively for POPD/Ppy-80/20, POPD/Ppy-50/50, POPD/Ppy-20/80.

### 3. Characterization

The viscosity average molar mass of homopolymers and copolymers were determined at room temperature using Ubbelhode Viscometer. FT-IR spectra of homopolymers and copolymers were taken on FT-IR spectrophotometer model Shimadzu IRA Affinity^−1^ in the form of KBr pellets. UV-visible spectra were taken on UV-visible spectrophotometer model Shimadzu UV-1800 in film form. The oscillator strength were calculated as reported in our earlier studies [30]. Fluorescence spectra of the sample were taken in on fluorescence spectrophotometer model Horiba Fluorolog@3. The quantum yield was calculated as per method reported in or earlier studies [8].

## 4. Result and discussions

### 4.1. Solubility and intrinsic viscosity studies

POPD was observed to be highly soluble in most of the polar solvents but Ppy was observed to insoluble, Table 1. With increasing OPD content, solubility of POPD. Ppy copolymer increased. POPD/Ppy-20/80 copolymer with the highest feed pyrrole content exhibited insolubility in common organic solvents. The copolymers having equal feed ratio of both monomers revealed partial solubility in all solvents. It was observed that enhancement of copolymer solubilty with increasing OPD content was attributed to higher solubulity of the later in common organic solvents [30,31].

**Table 1.** Solubility of homopolymers and copolymers in different solvents.

Polymer/copolymer	NMP	DMSO	THF	Methanol	Acetone
POPD	ES	ES	PS	PS	PS
Ppy	ES	PS	PS	IS	IS
POPD/Ppy-80/20	ES	PS	PS	PS	PS
POPD/Ppy-50/50	ES	PS	PS	PS	PS
POPD/Ppy-20/80	ES	PS	PS	IS	IS

Note:

(ES- Easily soluble; PS- partially soluble; ES-easily soluble; IS- insoluble).

**Figure 1.**
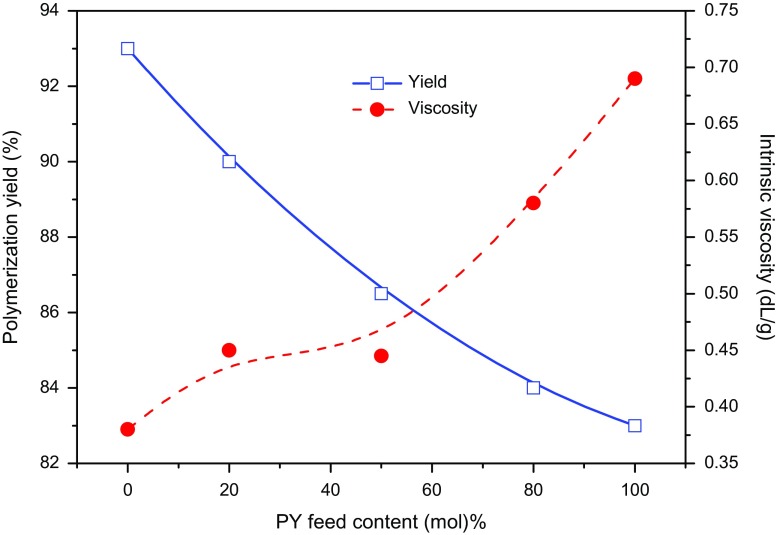
Influence of pyrrole (PY) feed content on polymerization yield and intrinsic viscosity of the copolymers.

The viscosity average molar mass was determined using 0.2 wt% solution of the homopolymers and copolymers in NMP using Ubbelhode Viscometer at room temperature and the intrinsic viscosities were determined using the Mark-Houwink equation [*η*] = *K M*_*v*_^a^, where *η* is intrinsic viscosity, [*η*] = 1.95 × 10^−6^*M*_*v*_^1.36^, *M*_*v*_ is viscosity average molar mass, *K* and *a* are Mark-Houwink constants [20]. PPy revealed highest molar mass due to the formation of a higher crosslinked structure [32], while POPD revealed viscosity average molar mass of 7841 Figure 1, Table 2. Intrinsic viscosities and viscosity average molar mass of the copolymers were found to be intermediate of homopolymers [32]. The low molar mass values indicated the formation of oligomeric structures.

**Table 2.** Intrinsic viscosities and viscosity average molar mass of synthesized polymers.

Homopolymer/copolymer	Intrinsic viscosity (*η*)	Viscosity average molar mass (Mv)
POPD	0.38	7841
Ppy	0.69	12280
POPD/Ppy-80/20	0.45	8999
POPD/Ppy-50/50	0.44	8869
POPD/Ppy-20/80	0.58	10846

The intrinsic viscosities and polymerization yield of the copolymers were inversely dependent on monomer mol ratio. The % yield decreased with an increase in feed ratio of pyrrole while the intrisic viscosity increased and reached up to 0.69 dL/g for the homopolymer. Enhancement of intrinsic viscosity suggested increament of molar mass along with rigidity of polymer chains [11]. It can therefore be concluded that intrinsic viscosities and yield of copolymers was influenced by the monomer feed ratio and confirmed the copolymerization.

### 4.2. FTIR studies

**Figure 2.**
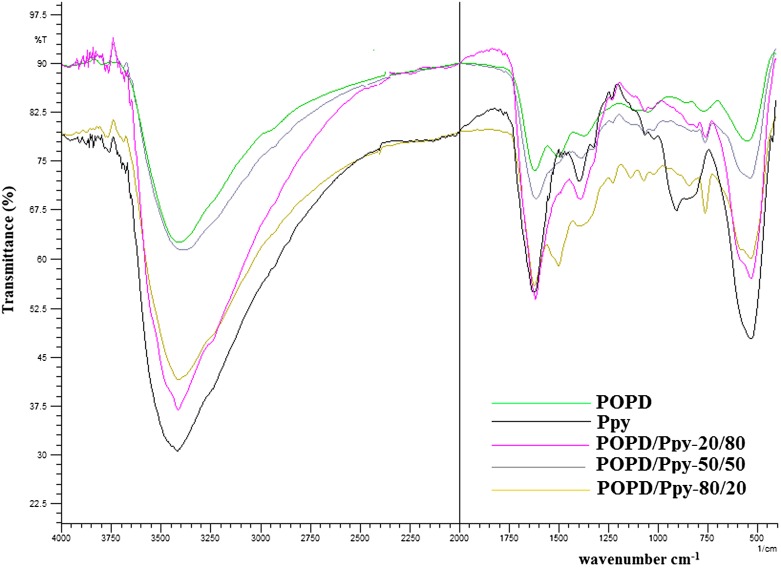
FTIR spectra of homopolymers and copolymers of POPD/Ppy.

The FTIR spectrum of POPD, Figure 2, revealed a peak at 3414 cm^−1^ due to the presence of secondary amino (-NH-) group while for Ppy, it was observed at 3421 cm^−1^. The imine stretching peak for POPD and Ppy were observed to be around 1618 and 1639 cm^−1^ respectively. In POPD peaks at 1500 and 1400 cm^−1^ were assigned to ring puckering of the quinonoid diamine and benzenoid diamine respectively while in case of Ppy, the peaks was observed at 1544 and 1400 cm^−1^ respectively. Benzenoid to quininoid ratio (*B/Q*) was calculated to be 0.93 for POPD and 0.89 for Ppy. The C-N stretching peak due to quininoid and benzenoid units was observed at 1310 and 1250 cm^−1^ for POPD, and at 1325 and 1251 cm^−1^ respectively for PPy. The peak at 850 cm^−1^ was recognized due to para substituted benzene while peak at 760 cm^−1^ was the characteristic peak of C-H out-of plane bending vibrations present on benzene nuclei in the phenazene skeleton in POPD while peak at 905 cm^−1^ attributed to C–H out-of-plane deformation vibrations of the ring in Ppy. The copolymer of POPD/Ppy-80/20 revealed NH stretching peak at 3410. The shifting of -NH peak to lower wavenumbers indicated electrostatic interaction of Ppy with POPD. Similarly NH stretching peak for POPD/Ppy-50/50 was found at 3387 cm^−1^ while for POPD/Ppy-20/80, it was noticed at 3420 cm^−1^. The copolymers containing higher content of Ppy revealed broad NH Peak similar to that of the homopolymer of Ppy while the copolymer containing higher POPD content showed similarity in the NH peak corresponding to the homopolymer of POPD. The benzenoid to quininoid (*B/Q*) was calculated as 0.90, 0.95 and 0.94 respectively in for POPD-Ppy-80/20, POPD-Ppy-50/50 and POPD-Ppy-20/80 indicating higher quininoid units present in copolymers.

The Fineman–Ross (FR) equation [32] was used to determine the reactivity ratios:

(1) $$F\left(f-1\right)/f=r^{1}\left(F^{2}/f\right)-r^{2}$$

where F = [OPD]/[PY] is the co-monomer feed ratio, r_1_ and r_2_ are reactivity ratios of the co-monomers, m_1_ and m_2_ are molar fractions of co-monomer units.

Copolymer composition was determined by analyzing the FTIR peak absorbance values corresponding to NH of POPD and NH of pyrrole according to the following equation:

(2) $$m_{1=}\frac{\Delta A^{763}/M_{1}}{\Delta A^{763}/M_{1}+\Delta A^{1400}/M_{2}}100$$

(3) $$m_{2=}\frac{\Delta A^{1400}/M_{2}}{\Delta A^{763}/M_{1}+\Delta A^{1400}/M_{2}}100$$

where M_1_ and M_2_ are molecular weights of OPD and PY units respectively. The ∆A^763^ corresponds to absorbance value of phenazine structure while ∆A^1400^ corresponds to absorbance value of the C–N pyrrole ring stretching vibration [33,34].

Results of the FTIR analyses of copolymers by using various initial monomer ratios are shown in Table 3. On the basis of these data, the values of absorption bands for the comonomer units are calculated which are used for the determination of copolymer compositions according to Equations (2) and (3). As evidenced from these data, with the change in the concentrations of OPD and Py units in the monomer feed from 80 to 20%, the formation of copolymers with almost identical molar ratios of m1/m2 monomer units was obtained. The feed ratio and the mol% ratio calculated from the FTIR data were found to be closely matching confirming that the copolymerization occurred as per the feed ratio. Monomer reactivity ratios were determined by Fineman-Ross plot of (F_2_/f) v/s [F(f-1)/f] for OPD and PY copolymers method and was found to be as *r*_1_ = −1.38 and *r*_2_ = 1.44 suggesting random copolymerization, Scheme 1.

**Table 3.** Monomer reactivity ratios and the Fineman Ross parameters of copolymers determined by FTIR analysis.

Monomer feed (mol %)		Monomer feed ratio	ΔA (OPD) unit	ΔA (PY) unit	Copolymer composition (mol %) by FTIR analysis		Unit ratio in copolymer	Parameters of Fineman Ross equation	
[OPD]	[PY]	F = [M_1_]/[M_2_]			m_1_	m_2_	*f* = *m*_1_*/m*_*2*_	F^2^/f	F(f-1)/f
80	20	4.000	0.120	0.018	84.60	15.38	5.50	2.9	3.27
50	50	1.000	0.580	0.496	40.41	56.46	0.71	1.4	−0.4
20	80	0.250	0.17	0.359	22.91	77.09	0.29	0.21	−0.5

### 4.3. UV-visible studies

**Figure 3.**
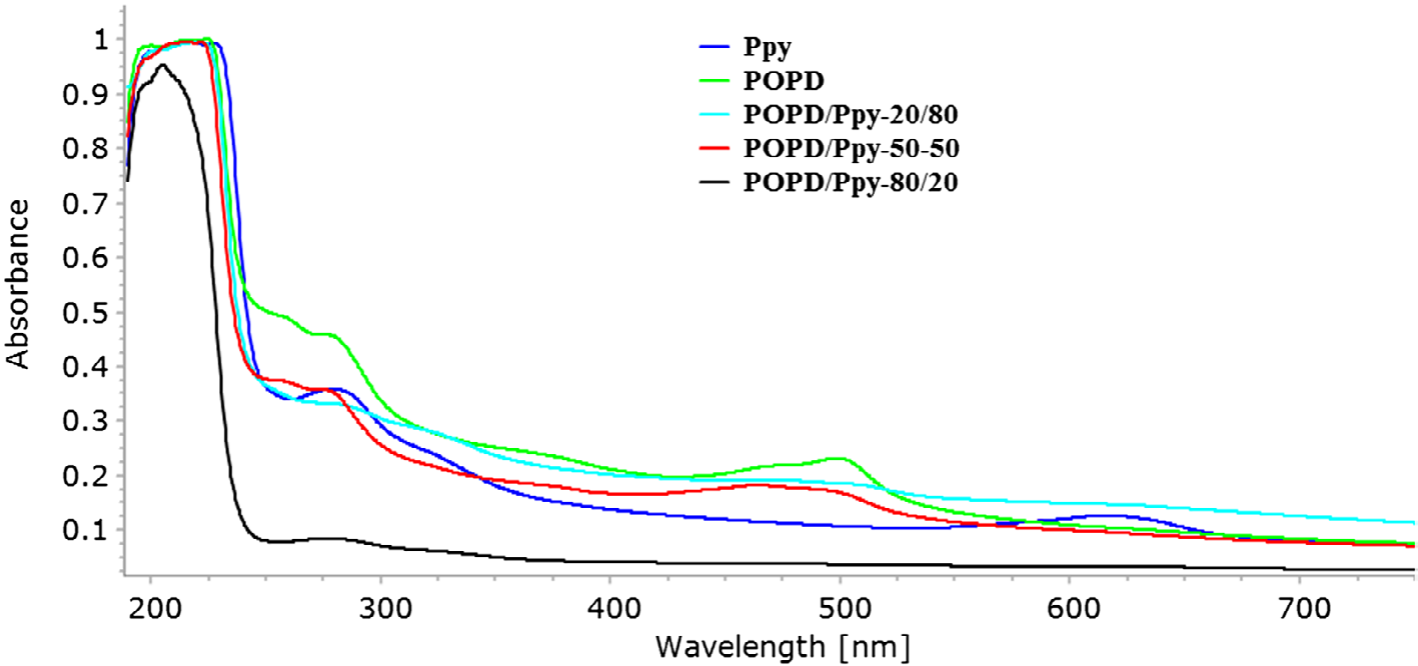
UV-visible spectra of POPD, Ppy and its copolymers.

UV-Visible spectra of POPD, Ppy and their copolymers are depicted in Figure 3. Pure POPD revealed peaks at 220, 277 and 500 nm in which were correlated with π- π* transitions and polaronic transition. Similarly Ppy revealed peaks at 220 and 620 nm; the later associated with doping of Ppy with MB dye thereby confirming the conducting state of polymer [5,30]. In the case of copolymers, the spectrum of POPD/Ppy-80/20 showed peaks at 480 nm (associated with POPD) which revealed a slight blue shift due to steric hindrance. The oscillator strength and molar extinction coefficient values were found to be higher for homopolymers as compared to the copolymers because of structural variation of both monomers. The oscillator strength values decreased in POPD/Ppy-80/20 and POPD/Ppy-20/80 for the peak at 460–475 as the o-phenylenediamine content decreased but increased for the peak observed around 620 nm with the increase in the pyrrole content, Table 4. The presence of the peaks associated with POPD and Ppy in the copolymers confirmed copolymerization of o-phenylenediamine with pyrrole.

**Table 4.** UV data of POPD, PPY and their copolymers.

Sample	*λ*_max_ (nm)	Molar extinction coefficient	Oscillator strength
POPD	474	28419	0.50
Ppy	612	24159	0.27
POPD/Ppy-80/20	471	25444	0.49
	630	14136	0.09
POPD/Ppy-50/50	471	4780	0.06
	620	4462	0.03
POPD/Ppy-20/80	471	32324	0.12
	620	28777	0.43

### 4.4. Fluorescence studies

**Figure 4.**
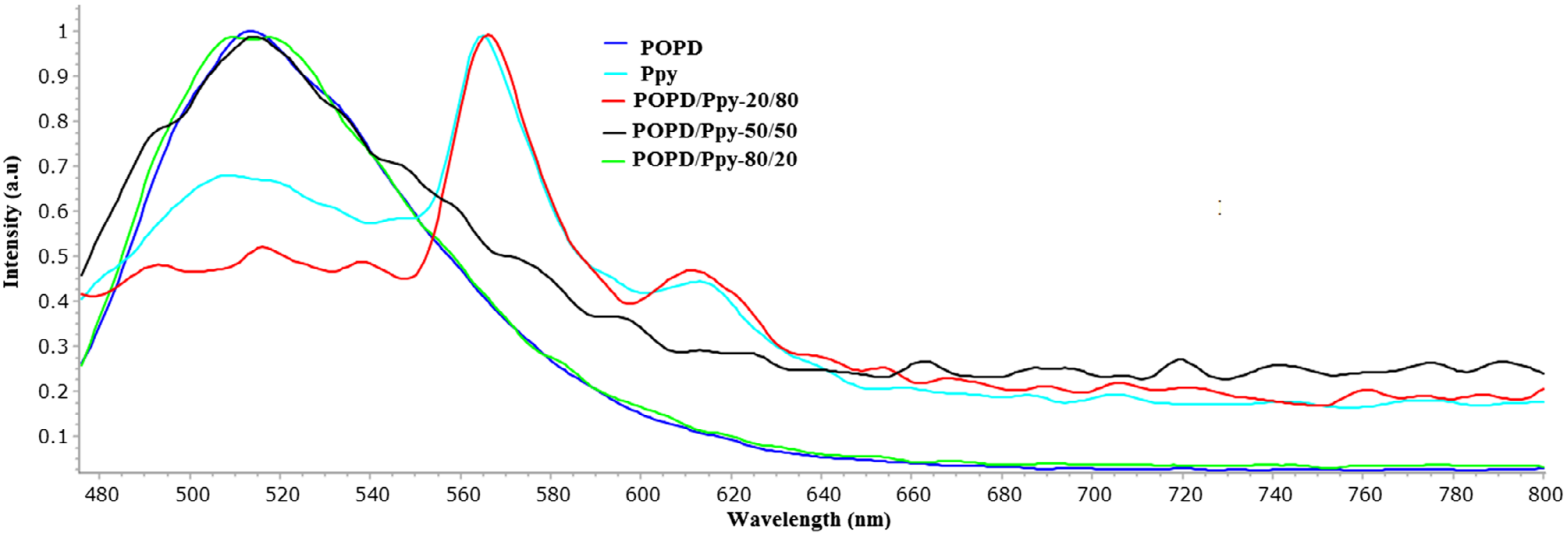
Fluorescence spectra of POPD, Ppy and their copolymers.

The fluorescence spectra of POPD, Ppy and their copolymers were excited at 450 nm and emission was recorded between 480 and 700 nm. In homopolymers emission spectrum of POPD, Figure 4 revealed a peak at 519 nm corresponding to the S_0_→S_1_ transition [5] while in Ppy there were two emission peaks were observed at 566 and 613 nm. The spectrum of copolymer, POPD/Ppy-80/20, Figure 4, exhibited peak at 518 nm. Among all three copolymers POPD/Ppy-50/50 showed peak at 518 nm. Quantum yield (ø) was calculated taking Rhodamine B as a reference material. Values of quantum yield for POPD, Ppy and for copolymers were calculated and they were observed 5.31 × 10^−3^, 3.81 × 10^−4^ for POPD and PPY, Table 5. Quantum yield values for copolymers were 4.70 × 10^−3^, 2.72 × 10^−3^, 2.27 × 10^−4^, respectively for POPD/PPy-80/20, POPD/PPy-50/50 and POPD/PPy-20/80. Values of (*ø*) were seen higher in which POPD content was higher. These results showed that the desired emission either in UV or visible range could be obtained by choosing the appropriate composition of the monomers in the copolymer.

**Table 5.** Fluorescence data of POPD, PPY and their copolymers.

Sample	*λ*_max_ (nm)	*A*_sample_	Integrated area	Quantum yield (ø)
POPD	519	0.72	7.58 × 10^7^	5.31 × 10^−3^
Ppy	566	0.44	3.33 × 10^6^	3.81 × 10^−4^
	613	0.51	7.11 × 10^5^	7.04 × 10^−5^
POPD/Ppy-80/20	518	0.55	5.18 × 10^7^	4.70 × 10^−3^
POPD/Ppy-50/50	515	0.136	7.36 × 10^6^	2.72 × 10^−3^
POPD/Ppy-20/80	567	0.65	2.94 × 10^6^	2.27 × 10^−4^
	611	0.68398	9.99 × 10^5^	7.34 × 10^−5^

The copolymers synthesized using MB dye as template medium was also found to act as a dopant for the copolymers thereby improving their optoelectronic properties. The electronic properties of the copolymer could therefore be controlled by varying the dye medium during synthesis as well as by altering co-monomer ratio in the copolymer. This technique of synthesizing doped copolymers is a facile method that could be utilized to tailor as well as tune the optoelectronic properties of conjugated polymers by choosing the composition of co monomers as per the desired potential application in designing LED devices, solar cells polymers, bioimaging etc.

## 5. Conclusion

Copolymerization of POPD and Ppy was carried for the systematic analysis of effect of copolymerization on the morphological, spectral and optical properties of conducting polymers. Viscosity average molar mass was determined in which molar mass of copolymers were varied between both the homopolymers that confirmed copolymerization. POPD/Ppy-50/50 exhibited least molar mass among all the three copolymers. FTIR analysis indicated the presence of both benzenoid and quinonoid structures and the quinonoid units were observed to higher than the benzenoid units. UV-Visible spectra showed variation in the polaronic peak absorbance behavior as the monomer feed ratio changed from POPD/PPY-80/20 to POPD/PPY-20/80 which reflected the influence of monomer functionality on the polaronic transitions. Fluorescence analysis confirmed that quantum yield was proportional to the amount of both monomers in the copolymers which could be tuned to obtained emission in the desired range.

## Disclosure statement

No potential conflict of interest was reported by the authors.

## Funding

This work was supported by Department of Science and Technology (DST), Science and Engineering Research Board (SERB) vide sanction [grant number SB/S1/PC-070/2013].
